# Supplementary material for: The relationship between egg size and helper number in cooperative breeders: a meta-analysis across species
Source: PeerJ. 2017 Nov 24;5:e4028. doi: 10.7717/peerj.4028 (PMC5704713; doi:10.7717/peerj.4028)
Supplement: Supplemental Information 1 [file peerj-05-4028-s001.docx]

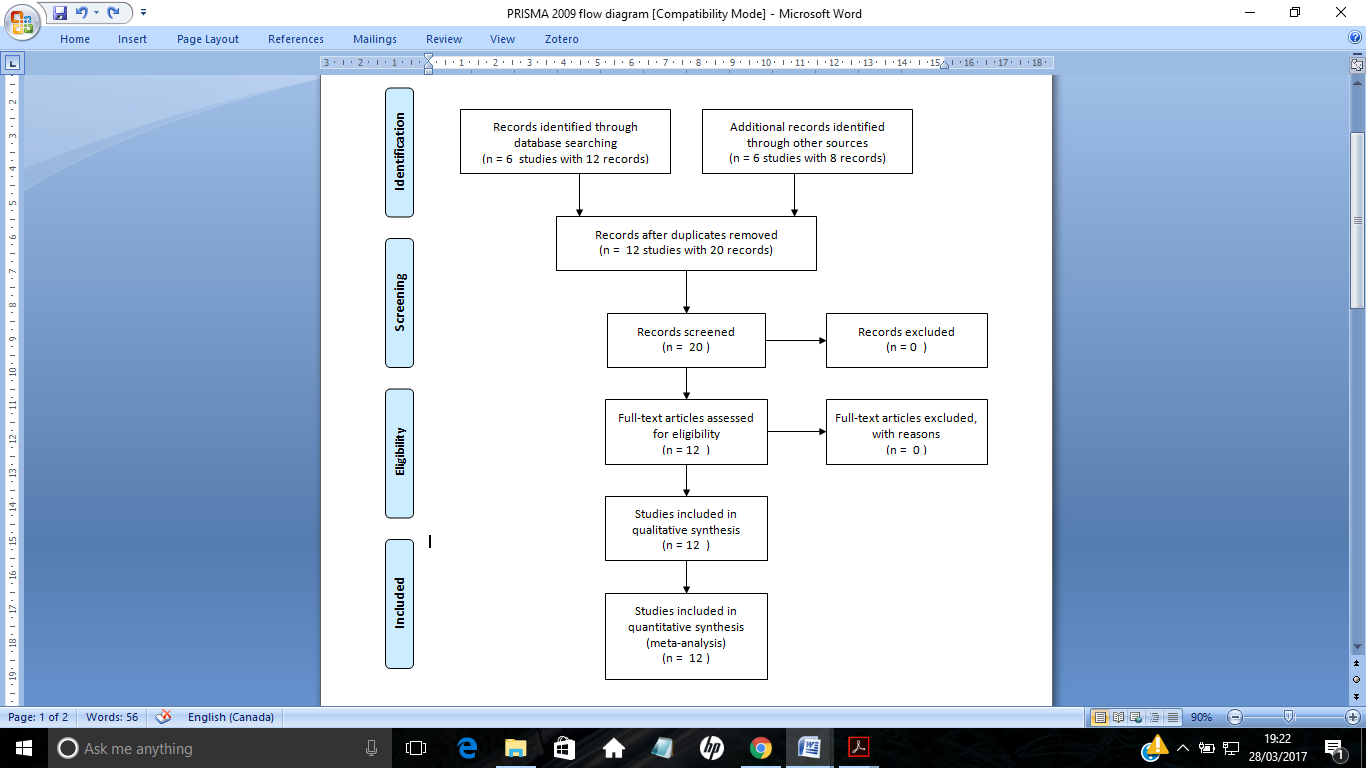


N.B. Due to the eligibility criteria being specific and decided in advance, no articles or records were excluded. For eligibility criteria and full details of record selection, please see methods section.

Supplementary Figure 1: A PRISMA flow diagram of the steps involved in data collection and analysis. Template from: Moher D, Liberati A, Tetzlaff J, Altman DG, The PRISMA Group (2009). Preferred Reporting Items for Systematic Reviews and Meta-Analyses: The PRISMA Statement. PLoS Med 6(7): e1000097. doi:10.1371/journal.pmed1000097
