# Supplementary material for: The relationship between egg size and helper number in cooperative breeders: a meta-analysis across species
Source: PeerJ. 2017 Nov 24;5:e4028. doi: 10.7717/peerj.4028 (PMC5704713; doi:10.7717/peerj.4028)
Supplement: Supplemental Information 6 [file peerj-05-4028-s006.docx]

**Rationale and contribution of meta-analysis**

In this study, we perform a meta-analysis to determine whether there is a general effect of number of helpers on egg size across cooperative breeders. We focused on maternal investment in egg size – which presumably cannot be directly influenced by helpers – rather than postnatal investment to avoid the confounding influence of the presence of helpers on traits indicating such investment, e.g. offspring size at weaning or fledging. Maternal plasticity in reproductive investment is expected to be more pronounced when helper efforts are additive (Hatchwell, 1999), while in cases where helpers adjust their levels of offspring care in response to the efforts of others any load-lightening appears to be shared such that mothers are not expected to reduce egg size (Langmore et al., 2016). A number of studies have recently started to provide the detailed information necessary to assess plasticity in egg investment traits in cooperative breeders, allowing us to assess whether there is general support for the load-lightening or the differential allocation hypotheses, as well as to determine whether the type of helper effort affects whether load-lightening or differential allocation occurs.

This meta-analysis contributes to the field of cooperative breeding and reproductive plasticity as it tests for the prevalence of two alternative maternal strategies, and has implications for our understanding of the benefits provided by helpers and whether these accrue primarily to breeders or offspring, the observation of concealed helper effects, and for understanding under which circumstances maternal investment should increase or decrease. There have been several empirical studies showing maternal investment varying with number of helpers (see references to Supplemental Table 1) but, to our knowledge, no meta-analysis testing for overall trends. We therefore provide the first meta-analysis in this field.
